# Addressing implicit bias and health disparities in a level IV NICU

**DOI:** 10.1038/s41372-023-01736-y

**Published:** 2023-07-28

**Authors:** Yolanda Brown-Madan, Amanda Williams, Seth Langston

**Affiliations:** Division of Neonatology, Department of Pediatrics, Cedars-Sinai Medical Center, Los Angeles, CA USA

**Keywords:** Paediatrics, Health occupations

## Introduction

An important factor in health inequity that has become increasingly prominent is the relationship between physician-patient racial discordance and the potential negative effect on healthcare outcomes. Importantly, these inequities trickle down to the youngest and most vulnerable of patients: newborns. Greenwood et al. demonstrated that among a cohort of over 1 million infants, the difference in mortality rate of Black newborns compared to White newborns was reduced by half when they received care by a Black physician, as compared to a race-discordant physician [1]. This alarming association may be secondary to implicit bias.

Education in health inequity and implicit bias may be instrumental in reducing inequities with the goal being to reduce an individual’s own biases. Implicit bias instruction has been shown to improve participants’ awareness and confidence in recognizing and managing implicit bias in healthcare [2]. To date, little has been studied regarding implicit bias training within the neonatal intensive care unit (NICU). The objective of our study was to design and implement small group workshops to provide experiential learning through role-play to highlight examples of implicit bias in the NICU.

## Methods

### Design

60 min small-group workshops discussing health inequities and implicit biases were implemented for NICU nurses in a level IV NICU. The workshop leaders were a Neonatologist and the NICU Clinical Nurse Specialist. Prior to beginning each workshop, the participants completed an electronic survey requesting demographic data and participants self-reported understanding of health disparities. All questions were in Likert scale format with answers ranging from ‘strongly agree’ to ‘strongly disagree.’ Each session started with an introduction to health disparities and implicit bias which included a review of recent literature and feedback gathered from a cohort of Black mother’s past experiences in the CSMC NICU.

A novel NICU approach involving role playing was subsequently used to demonstrate examples of implicit bias. Although our study shares similarities with simulation, we describe our approach as role-playing as the learners were observers and not active participants in the scenarios. Our team intentionally provided role playing simulations with small groups to allow for psychological safety and active participation among the attendees.

Debriefing and self-reflection ensued after each role-playing scenario, in which the learners were active participants. On conclusion of each workshop, the participants completed another survey that summarized their experience.

To measure the effect of our workshops, we distributed a six-month follow-up survey to inquire how the participants’ experience has influenced their ongoing considerations of health inequities and the longstanding value of the workshop.

## Results

A total of 95 participants completed the in-person workshops with the majority identifying as ‘female’ between the ages of 20 and 40 (Table 1). Participant agreement includes those that ‘strongly agree’ and ‘agree.’ Pre-survey results showed 91% of participants believed workshops regarding racial equity are valuable with 77% believing race impacts a neonate’s health outcome. Overall, 96% of participants agreed there is a benefit in discussing the impact of race on health outcomes in the NICU. Post-survey results (Table 2) revealed that 99% believed the workshop to be a valuable learning experience with a preference (94%) for in-person learning as compared to virtual options. Importantly, 97% identified increased mindfulness of the patient experience based on race and socioeconomic background with an overwhelming majority of participants interested in continued education in health inequities. The 6-month follow-up survey showed 99% of respondents felt there continued to be value in discussing the impacts of race on our work in the NICU.

**Table 1:** Demographics of study participants.

Variable	*N* = 95 (%)
Age Groups (yrs)	
20–30	30 (32)
31–40	29 (31)
41–50	16 (17)
51–60	15 (15)
>60	5 (5)
Race	
Caucasian	26 (27)
Black or African American	9 (10)
Hispanic or Latino	11 (12)
Asian or Pacific Islander	45 (47)
Other^a^	4 (4)
Gender	
Male	4 (4)
Female	90 (95)
Non-binary	1 (1)
Number of Health Disparities Workshops attended in past 2 years	
None	26 (27)
1–2	58 (61)
3 or more	11 (12)

*N* Number, *yrs* years.

^a^Other race includes Native American, Other, Mixed.

**Table 2:** Post-workshop survey results.

Statement	Strongly Agree	Somewhat Agree	Neutral	Somewhat Disagree	Strongly Disagree
The Health Disparities Workshop was a valuable learning experience	83 (87%)	11 (12%)	0 (0%)	0 (0%)	1 (1%)
I prefer in person workshops discussing health disparities instead of virtual (online) programs	77 (81%)	12 (13%)	6 (6%)	0 (0%)	0 (0%)
This workshop provided me with resources to promote health equity	69 (73%)	24 (25%)	0 (0%)	1 (1%)	1 (1%)
This workshop has allowed me to identify personal bias or misconceptions regarding health inequality	70 (74%)	22 (23%)	2 (2%)	0 (0%)	1 (1%)
This workshop has encouraged me to be more mindful of differences in patient’s experiences and social demographic backgrounds	82 (86%)	12 (13%)	1 (1%)	0 (0%)	0 (0%)

Data are shown as numbers (percentages).

## Discussion

Implicit bias has been shown to cause medical professionals to disregard mothers who are Black and/or economically disadvantaged, which is related to racial inequities in maternal and infant morbidity and mortality that led to many of the current laws mandating implicit bias training [3, 4].

Specific implicit bias curriculum that focuses on the field of neonatal-perinatal medicine is limited. Our study highlights the importance of small group in-person workshops as a tool of implicit bias training in the NICU, as well as the feasibility in implementing these workshops to actively address health inequities. Importantly, participants preferred in-person workshops to virtual learning allowing for active learning.

Our study was not without limitations including being a single-center study and lack of formal training in diversity, equity, and inclusion for session leaders, which has been recommended for implicit bias simulation training [5]. We believe the strengths of our study included utilization of small group, interactive sessions where participants engaged in an active learning process.

One of the fundamental parts of implicit bias training is to assist in recognizing the individual’s own biases and how to address observed inequities in the workplace. At the conclusion of our session, the post-survey revealed >95% of participants were able to identify their own personal bias or misconceptions regarding health inequality.

### Supplementary information

Supplemental Material. Pre-workshop survey results.
