# Correction: Addressing implicit bias and health disparities in a level IV NICU

**DOI:** 10.1038/s41372-023-01768-4

**Published:** 2023-09-18

**Authors:** Yolanda Brown-Madan, Amanda Williams, Seth Langston

**Affiliations:** Division of Neonatology, Department of Pediatrics, Cedars-Sinai Medical Center, Los Angeles, CA USA

**Keywords:** Paediatrics, Health occupations

Correction to: *Journal of Perinatology* 10.1038/s41372-023-01736-y, published online 28 July 2023

In this paper, in the Introduction paragraph, the explanation of the Greenwood et al. citation has been revised. It now reads as follows:

Greenwood et al. demonstrated that among a cohort of over 1 million infants, the difference in mortality rate of Black newborns compared to White newborns was reduced by half when they received care by a Black physician, as compared to a race-discordant physician.

The original article has been corrected.

