# Supplementary material for: Addressing implicit bias and health disparities in a level IV NICU
Source: J Perinatol. 2023 Jul 28;43(12):1494–6. doi: 10.1038/s41372-023-01736-y (PMC10716036; doi:10.1038/s41372-023-01736-y)
Supplement: Supplementary file 1 — Supplemental Material. Pre-workshop survey results. [file 41372_2023_1736_MOESM1_ESM.docx]

**Supplemental Material. Pre-workshop survey results.**

| **Statement** | **Strongly Agree** | **Somewhat Agree** | **Neutral** | **Somewhat Disagree** | **Strongly Disagree** |
| --- | --- | --- | --- | --- | --- |
| **Workshops about racial equity are useful** | 50 (53%) | 36 (38%) | 4 (4%) | 2 (2%) | 3 (3%) |
| **I feel comfortable talking about race** | 43 (45%) | 44 (46%) | 1 (1%) | 7 (8%) | 0 (0%) |
| **I am comfortable when others talk about race** | 51 (54%) | 40 (42%) | 1 (1%) | 3 (3%) | 0 (0%) |
| **The NICU is a diverse and inclusive work environment** | 47 (49%) | 41 (43%) | 0 (0%) | 5 (6%) | 2 (2%) |
| **I am comfortable asking our patient's parents about their own racial identification** | 58 (61%) | 29 (31%) | 1 (1%) | 6 (6%) | 1 (1%) |
| **I think a patient's race impacts their health outcomes** | 43 (45%) | 30 (32%) | 6 (6%) | 12 (13%) | 4 (4%) |
| **I am aware of a patient's race when caring for them** | 32 (34%) | 48 (50%) | 2 (2%) | 12 (13%) | 1 (1%) |
| **I am aware if a patient's race is different from my own** | 51 (54%) | 35 (37%) | 0 (0%) | 8 (8%) | 1 (1%) |
| **I think it's valuable to examine and discuss the impacts of race on our work** | 72 (76%) | 19 (20%) | 1 (1%) | 2 (2%) | 1 (1%) |
| **I think trainings/workshops on racial equity and inclusion will improve patient outcomes in the NICU** | 60 (63%) | 24 (24%) | 5 (6%) | 5 (6%) | 1 (1%) |

Abbreviations

NICU, Neonatal Intensive Care Unit

Data are shown as numbers (percentages).
